# Identification of Novel *NSD1* variations in four Pediatric cases with sotos Syndrome

**DOI:** 10.1186/s12920-024-01889-5

**Published:** 2024-04-29

**Authors:** Zhuo Ren, Ling Yue, Hua-ying Hu, Xiao-lin Hou, Wen-qi Chen, Ya Tan, Zhe Dong, Jing Zhang

**Affiliations:** 1 Department of Obstetrics and Gynecology, Peking University International Hospital, Beijing, China; 2 Department of Pediatric Neurology Rehabilitation, Hebei Children’s Hospital, Shijiazhuang, Hebei China; 3 Birth Defects Prevention and Control Technology Research Center, Medical Innovation Research Division of Chinese PLA General Hospital, Beijing, China; 4 Prenatal Diagnosis Center, Hebei Key Laboratory of Maternal and Fetal Medicine, Shijiazhuang Key Laboratory of Reproductive Health, Shijiazhuang Obstetrics and Gynecology Hospital, 16 Tangu-North Street, Shijiazhuang, Hebei China

**Keywords:** SOTOS syndrome, *NSD1*, Overgrowth, Whole exome sequencing, Structural analyses

## Abstract

**Objective:**

Sotos syndrome (SOTOS) is an uncommon genetic condition that manifests itself with the following distinctive features: prenatal overgrowth, facial abnormalities, and intellectual disability. This disorder is often associated with haploinsufficiency of the nuclear receptor-binding SET domain protein 1 (*NSD1*)gene. We investigated four pediatric cases characterized by early-onset overgrowth and developmental delay. The primary objective of this study was to achieve accurate genetic diagnoses.

**Design&Methods:**

A sequential analysis approach comprising chromosomal karyotyping, whole exome sequencing, and microarray analysis was conducted.

**Results:**

All four cases exhibited variations in the NSD1 gene, with the identification of four previously unreported de novo variants, each specific to one case.Specifically, Case 1 carried the *NSD1* (NM_022455): c.2686 C > T(p.Q896X) variant, Case 2 had the *NSD1* (NM_022455): c.2858_2859delCT(p.S953X) variant, Case 3 displayed a chromosomal aberration, chr5: 5q35.2q35.3(176,516,604–176,639,249)×1, which encompassed the 5′-untranslated region of *NSD1*, and Case 4 harbored the *NSD1* (NM_022455): c.6397T > G(p.C2133G) variant.

**Conclusion:**

This study not only provided precise diagnoses for these cases but also supplied significant evidence to facilitate informed consultations. Furthermore, our findings expanded the spectrum of mutations associated with SOTOS.

**Supplementary Information:**

The online version contains supplementary material available at 10.1186/s12920-024-01889-5.

## Introduction

Sotos syndrome (SOTOS), previously known as cerebral gigantism (OMIM #117,550), was first described by Juan Fernandez Sotos et al. in 1964 [1]. This neurologic disease is characterized by excessive growth from the prenatal stage to childhood, accompanied with distinctive facial abnormalities (large skull, pointed chin, and acromegaly), advanced bone age, seizures, occasional brain abnormalities, and intellectual disability [2, 3]. The estimated prevalence of SOTOS is 1 in 14,000 live births. The prominent features include behavioral abnormalities, often within the autistic spectrum, abnormal cranial magnetic resonance imaging (MRI)/computed tomography (CT) findings, cardiac anomalies, joint hyperlaxity with or without pes planus, neonatal complications, maternal preeclampsia, scoliosis, renal anomalies, and seizures [2]. However, the definitive clinical diagnostic criteria of SOTOS has been challenging because its characteristic features may be potentially misdiagnosed for other genetic disorders, such as Weaver–Smith syndrome (OMIM #277,590) [4], Malan syndrome (OMIM #614,753) [5], Cohen–Gibson syndrome (OMIM #617,561) [6], and other similar disorders [7]. Thus, genetic techniques are considered crucial for the accurate differential diagnosis of these conditions.

SOTOS follows an autosomal dominant inheritance pattern with complete penetrance, and > 95% of the affected individuals carry a *de novo* pathogenic variant [2]. The nuclear receptor-binding SET domain protein 1 (*NSD1*) gene (OMIM *606,681), located on chromosome 5q35.3 and containing 23 exons, is the only gene that has been identified to cause SOTOS [8]. The diagnosis of SOTOS in the proband can be achieved by identifying either a heterozygous pathogenic (or potentially pathogenic) variant in *NSD1* or a deletion encompassing *NSD1* through molecular genetic testing [3, 9, 10]. So far,>500 disease-causing variants have been documented and indexed in the Human Gene Mutation Database (https://www.hgmd.cf.ac.uk/; 20,230,801). These variants encompass a diverse spectrum of mutations, including missense, truncating, partial gene deletions, splice site alterations, and 5q35 microdeletions, and all of them lead to *NSD1* haploinsufficiency. The past two decades have seen significant advancements in genetic technology with notable enhancements in the detection efficiency and classification accuracy of SOTOS-related variations [11]. While the genotype–phenotype correlation of SOTOS has been explored in a few studies [3, 9, 12–14], the consensus is that individuals carrying the 5q35 microdeletion have milder overgrowth but more severe intellectual disabilities than those with the intragenic *NSD1* pathogenic variant [2]. However, several other symptoms lack well-established associations with specific variants, necessitating further investigation.

The present study presents clinical and genetic findings, as well as imaging data of four pediatric cases diagnosed with SOTOS. The identification of the novel *NSD1* variants in these cases not only contributes to a more comprehensive understanding of the condition but also expands the mutation spectrum associated with SOTOS.

## Materials and methods

This study obtained approval from the Ethics Committee of Shijiazhuang Obstetrics and Gynecology Hospital (approval no. 20,230,160). Informed consent was acquired from the legal guardians of all minor participants. All procedures conducted in this study adhered to the principles outlined in the Declaration of Helsinki of 1964, along with its subsequent revisions and pertinent ethical standards.

### Participants and clinical assessment

Four cases of suspected SOTOS fetuses were admitted to our center between December 2020 and January 2023. A comprehensive clinical assessment comprising general clinic monitoring in the outpatient department and an MRI examination, followed by a thorough investigation of the family history, was conducted.

### Karyotyping and copy number variation analyses

A comprehensive genetic diagnosis was performed with peripheral blood (PB) samples collected from the probands for traditional chromosomal karyotyping using G-binding for total chromosomal anomalies detection [15].

Genomic DNA extraction was performed with the PB samples collected from the probands and parents using the QIAamp DNA Midi Kit (Qiagen, Germany). Chromosomal microarray analysis (CMA) was conducted using the CytoScan 750 K SNP-array (Affymetrix Inc., USA) following manufacturer’s protocols to investigate clinically significant genomic copy number variations (CNVs).

### Whole exome sequencing

Sequence variants were detected in the proband samples using whole exome sequencing (WES), in accordance with our previously described method [16].Briefly, Agilent Sure Select Human Exon Sequence Capture Kit (Agilent, USA) was used for the enrichment of target region sequences. Quantitative polymerase chain reaction (qPCR) and Agilent Bioanalyzer 2100 (Agilent, USA) were utilized to assess the DNA library quality by determining the size, content, and distribution. Subsequently, the NovaSeq6000 platform (Illumina) was used to sequence DNA samples having ?∼ 150 bp paired-end reads, with approximately 300 pM per sample using the NovaSeq Reagent kit. The sequenced raw reads were aligned to the human reference genome (accession no.: hg19/GRCh37) using the Burrows Wheeler Aligner tool(quality level Q30% > 90% and the quality criteria are listed at https://www.illumina.com/science/technology/next-generation-sequencing/plan-experiments/quality-scores.html).Picard(v1.57) Variant calling was employed for the purpose of removing PCR duplicates using the Genome Analysis Tool Kit (https://software.broadinstitute.org/gatk/) and the Verita Trekker® Variants Detection system (v2.0; Berry Genomics, China). This process was performed using the ANNOVAR (v2.0) and Enliven® Variants Annotation Interpretation systems (Berry Genomics [17], and the guidelines recommended by the American College of Medical Genetics and Genomics(ACMG) were followed [18]. For variant pathogenicity interpretation, three frequency databases (ExAC_EAS, http://exac.broadinstitute.org; gnomAD_exome_EAS, http://gnomad.broadinstitute.org; 1000G_2015aug_eas, https://www.internationalgenome.org), and Human Gene Mutation Database (pro v2019) were used as reference. Additionally, the REVEL (rare exome variant ensemble learner; an integrated pathogenicity prediction approach) [19] and the pLI (probability of being loss-of-function intolerant; indicating tolerance of truncating variants) scores were used.

The sequence verification was performed with Sanger sequencing using the 3500DX Genetic Analyzer (Applied Biosystems, Thermo Fisher Scientific, USA). Quantitative fluorescence PCR (qfPCR) was employed to confirm the CNV results (detailed method described in Supplementary Material 1).

### Conservatism and structural analyses

The evolutionary conservation of affected amino acid residues related to the respective missense variant was analyzed using MEGA 7 (http://www.megasoftware.net/previousVersions.php) with default parameters. Structure models for the wildtype and missense variant were constructed using the AF-Q96L73-F1 structure prediction (https://alphafold.ebi.ac.uk/entry/Q96L73) with the Swiss-Model program (https://swissmodel.expasy.org/)including default parameters.

## Results

### Clinical presentation

**Fig. 1:**
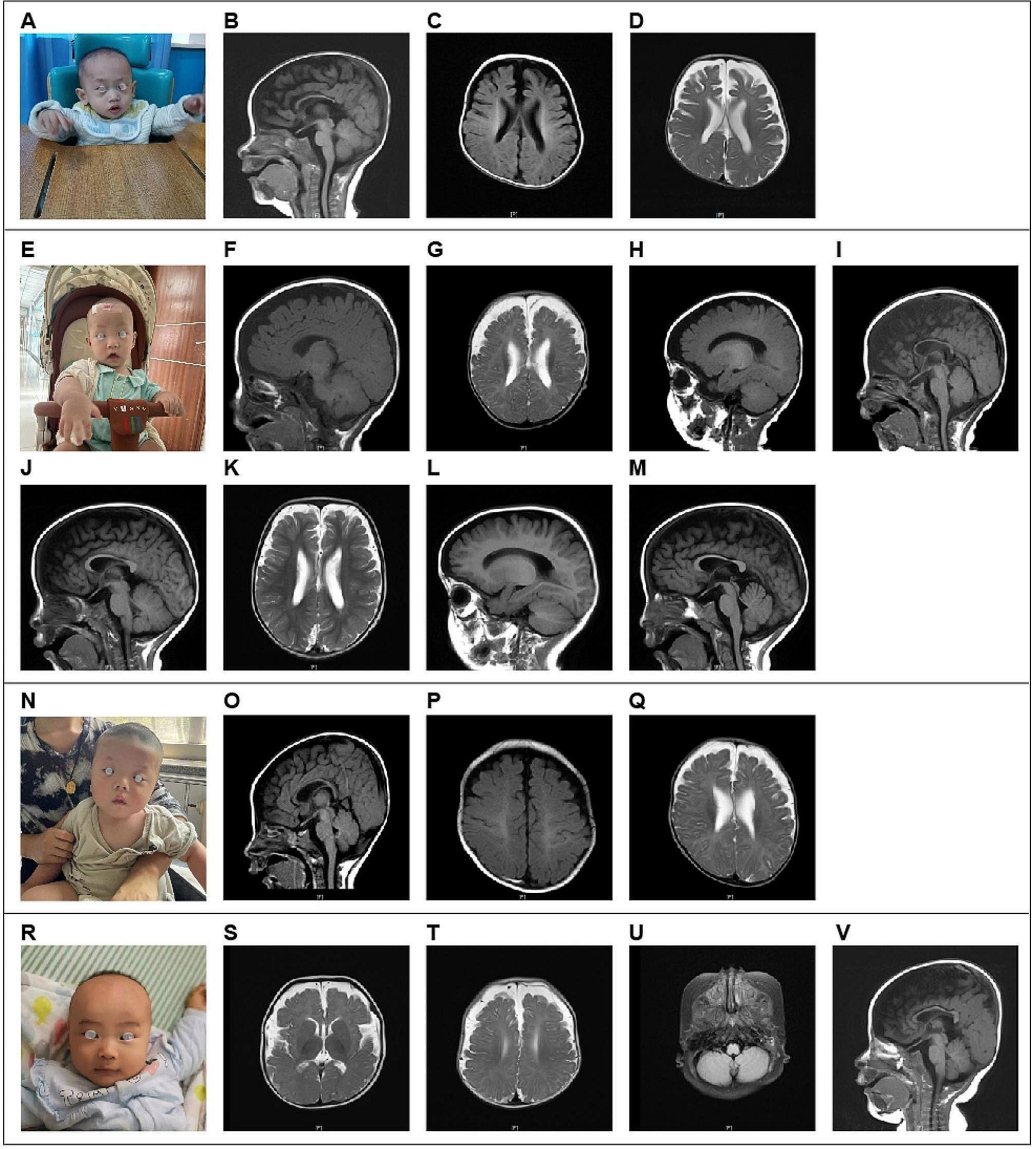
MRI indications in cerebral magnetic resonance imaging for the four cases. **A**–**D**: Case 1 had delayed myelination, widening of the bilateral ventricles, and dysplasia of the corpus callosum. **E**–**I**: (4 months and 9 days): Case 2 possessed a smaller brain mass, exhibited widening of the bilateral ventricles, and incomplete closure of the transparent compartments; the cerebral sulcus was slightly wider, the bifrontotemporal extracerebral space was significantly wider, and the corpus callosum was narrow; and a long T2 signal occurred in the bilateral mastoid; **J**–**M**: (1 year and 4 months): In Case 2, a reduction in the widening of the bifrontotemporal extraencephalic space and an increased in the widening of the bilateral ventricles was noted. **N**–**Q**: Case 3 had slightly delayed myelination, widening of the bifrontotemporal extracerebral space, irregular widening of the bilateral ventricles, and slightly thinner corpus callosum. **R**–**V**: Case 4 had a smaller frontal lobe volume, an old bleeding lesion in the left occipital lobe, a softened lesion with hemosiderosis, low signal in the right cerebellar hemisphere, significant widening of the bilateral frontotemporal extracerebral space, and thin corpus callosum

The four cases featured in this study underwent initial assessment before reaching the age of 2 years, with the earliest case observed at just 4 months of age. Case 1, who underwent the initial evaluation at 1 year and 11 months, presented with impaired language and cognitive development and displayed unsteady walking. This individual was delivered via cesarean section at full term and encountered oxygen deprivation during birth. The physical examination unveiled several notable characteristics, including a head circumference measuring 50 cm (approximately at the 95th centile), a distinctive facial appearance marked by a wide interocular distance and prominent forehead, above-average height, and lags in response. The patient’s posture exhibited atypical characteristics, including hip flexion, strephexopodia, and a tendency to experience falls. Fine motor skills of both hands were notably restricted, with limited finger pinch flexibility. His responsiveness to commands was inadequate, and he encountered challenges in feature recognition. Cardiac ultrasound examination unveiled the presence of an atrial septal defect, while cerebral MRI revealed delayed myelination, bilateral ventricular enlargement, and dysplasia of the corpus callosum (Fig. 1A-D).

Case, a four-month-old male infant, presented at our outpatient clinic with complaints of head instability and difficulty in turning over. During the physical examination, it was noted that he had a head circumference measuring 47 cm (above the 99th centile), wide interocular distance, and ears set lower than usual. Furthermore, he displayed delayed responses to both sounds and objects. His Albert Motor Scale score was 35 (equivalent to the 6th month, at the 10th–25th centile, indicating low-to-medium motor development). Cardiac ultrasound examination revealed a loose and thickened left ventricular apex myocardial tissue structure, along with mild regurgitation at the bicuspid and tricuspid valves. Cerebral MRI findings in Case 2 indicated delayed myelination, substantial enlargement of the bifrontal extracerebral space and bilateral ventricles, as well as thinning of the corpus callosum, collectively suggestive of brain dysplasia(Figs. 1E–M).

Moving on to Case 3, a 5-month-old female infant, she was also referred to our outpatient clinic due to head instability and difficulty in turning over. Her physical examination revealed a head circumference measuring 47.5 cm (above the 99th centile), wide-set eyes, a pointed chin, ears set lower than typical, and a high-arched palate. Additionally, she exhibited delayed responses to sounds and objects and had elevated muscle tone in her lower limbs. Cardiac ultrasound revealed the presence of an atrial septal defect and an increased forward velocity of the pulmonary valve. Cerebral MRI demonstrated delayed myelination, a small subdural hemorrhage in the right parietal lobe, widening of the extracerebral space in the bilateral frontotemporal region, irregular enlargement of the bilateral ventricles, and a slightly thinner corpus callosum(Figs. 1 N–Q).

Case, a 5-month-old girl, visited our clinic for delayed motor development. Physical examination revealed almost similar manifestations as those observed in Case 3 (head circumference of 47.5 cm, wide-set eyes, pointed chin, low-set ears, and high-arched palate). Cardiac ultrasound revealed an atrial septal defect and increased forward velocity of the pulmonary valve. Cerebral MRI demonstrated delayed myelination, a small amount of subdural hemorrhage in the right parietal lobe, widening of the bilateral frontotemporal extracerebral space, irregular widening of the bilateral ventricles, and slightly thinner callosum (Fig. 1R–V).

### Genetic assessment

**Fig. 2:**
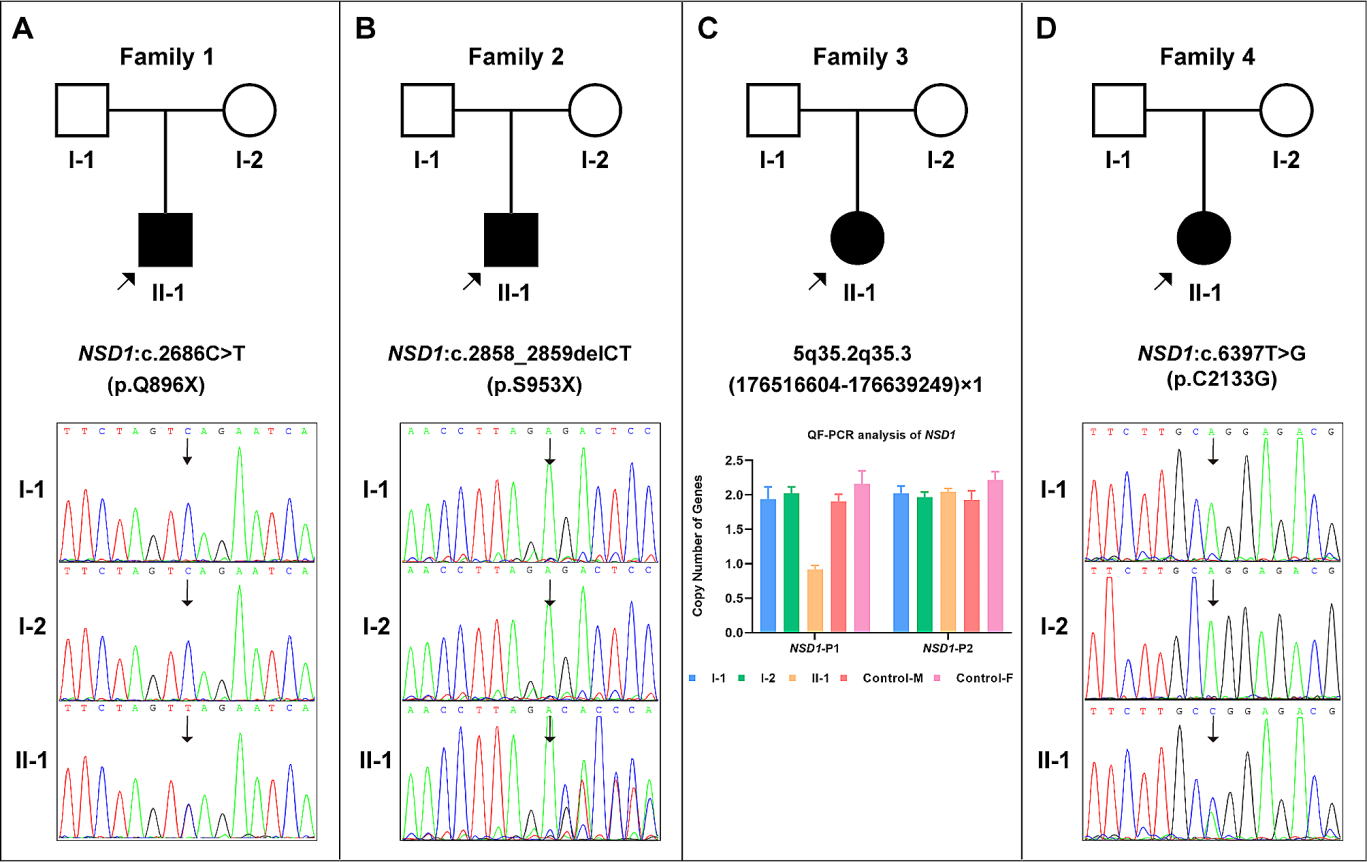
Genetic findings for the four probands. **A**: Case 1, *de novo NSD1* (NM_022455): c.2686 C > T(p.Q896X) variant. **B**:Case 2, *de novo NSD1* (NM_022455): c.2858_2859delCT(p.S953X) variant. **C**: Case 3, *de novo* heterozygous microdeletion, chr5: 5q35.2q35.3(176,516,604–176,639,249)×1 (*NSD1*-P1), containing the 5′-UTR region of *NSD1*; the result on the right side (*NSD1*-P2, chr5:177,235,826–177,235,945) suggests that the coding area of *NSD1* was normal. **D**: Case 4, *de novo NSD1* (NM_022455): c.6397T > G(p.C2133G) variant

The pedigree diagram for each case is shown in Fig. 2. Karyotyping analysis results were normal for all four cases. In Case 3, CMA detected a *de novo* heterozygous microdeletion, chr5: 5q35.2q35.3(176,516,604–176,639,249)×1, encompassing the 5′-UTR fragment of NSD1, which was subsequently confirmed using qfPCR (Fig. 2C). No abnormalities in the CMA results were observed in the remaining three cases.

WES successfully identified the causative variants in the remaining three cases, and the results were validated with Sanger sequencing. Specifically, Case 1 carried a *de novo* truncating variant, *NSD1*(NM_022455): c.2686 C > T(p.Q896X) (Fig. 2A); Case 2 harbored a *de novo* frame-shift variant, *NSD1*(NM_022455): c.2858_2859delCT(p.S953X), resulting in early translation termination (Fig. 2B); and Case 4 exhibited a *de novo* missense variant, *NSD1*(NM_022455): c.6397T > G(p.C2133G)(Fig. 2D). All the three sequence variations identified were novel and have not been previously reported.

The details of the identified variations in the four cases are presented in Table 1.

### Missense variant analyses

**Fig. 3:**
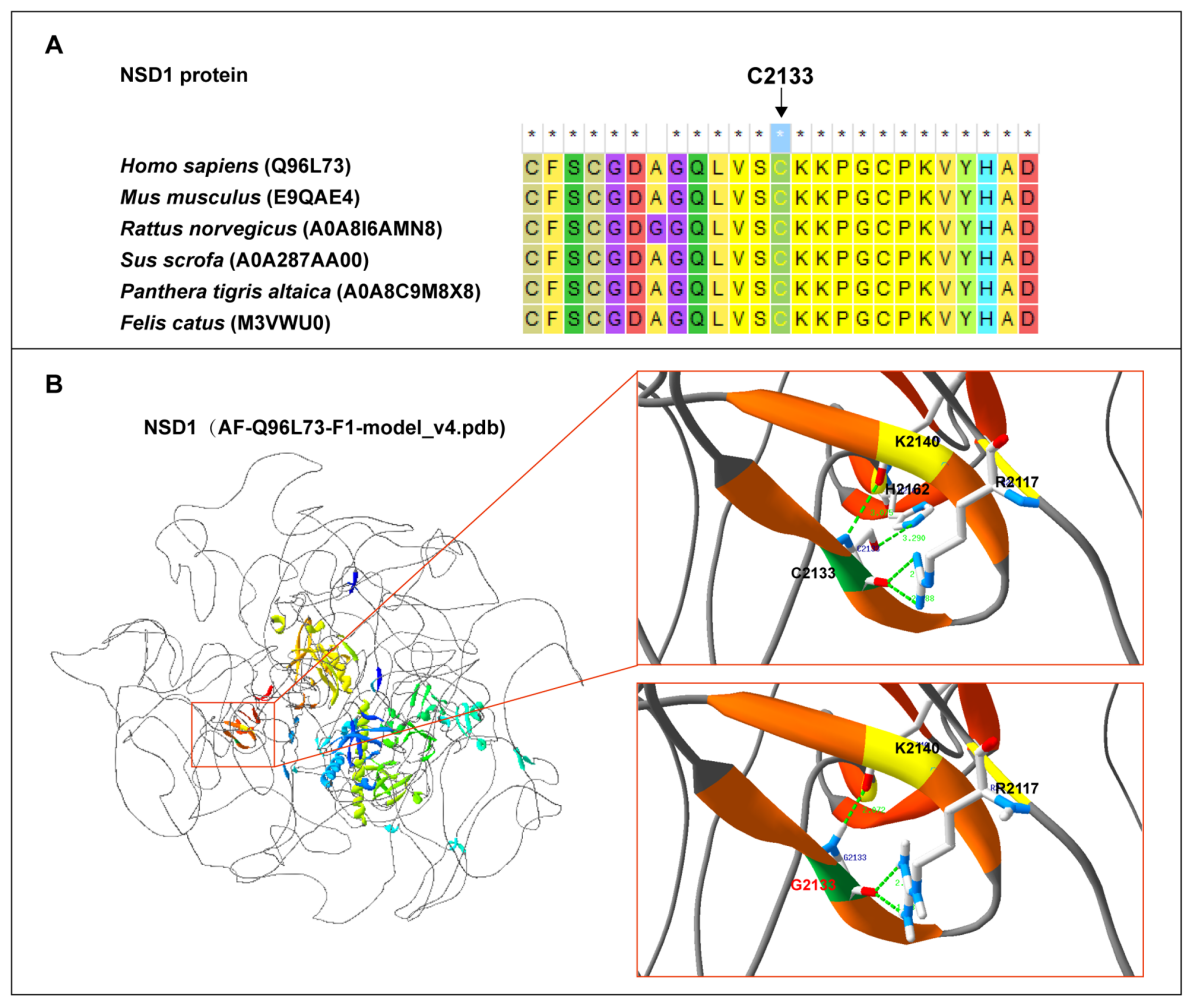
Analysis of the missense variant. **A**: The C2133 residue is conserved across species. **B**: NSD1: p.C2133G variation significantly affected hydrogen bond formation at this location

We also identified a solitary missense variant, *NSD1*(NM_022455): c.6397T > G(p.C2133G). According to MEGA 7 results, the p.C2133 residue was conserved across multiple species (Fig. 3A). Structural modeling analysis revealed that p.C2133G primarily affected local hydrogen bond formation. In the wildtype model, the C2133 site formed hydrogen bonds with K2140, R2117, and H2162 within the β-helix. The interactions of C2133 with K2140 and H2162 occurred inside the β-helix, and the interactions between C2133 and R2117 occurred outside the β-helix (Fig. 3B, upper right). In the missense variant model, C2133G exhibited hydrogen bonds with K2140 and R2117,but it was absent between G2133 and H2162. Additionally, the distance of the hydrogen bonds formed between G2133 and K2140 as well as G2133 and R2117 was altered (Fig. 3B, lower right).

## Discussion

Neonatal overgrowth is a common clinical concern with a multifaceted etiology. It can result from various factors, including maternal lifestyle choices during pregnancy, gestational diabetes, neonatal hyperinsulinism, or congenital syndromes [20]. Overgrowth syndromes encompass a diverse array of rare conditions characterized by either segmental or generalized overgrowth, often accompanied by additional features such as macrocephaly, visceromegaly, and other symptoms [7, 21]. Recent advancements in molecular technology, particularly next-generation sequencing (NGS), have significantly improved the efficiency of identifying novel causative genes and have increased the detection rate for overgrowth syndromes.

SOTOS represents a distinctive form of overgrowth primarily involving nervous system development. Many patients with SOTOS exhibit varying degrees of learning disabilities, ranging from mild to severe, along with behavioral abnormalities and symptoms related to autism spectrum disorder [22]. More than 40% of SOTOS cases are accompanied with seizures [3]. Skeletal manifestations in this disorder are noted, such as scoliosis(50%), along with flat feet and genu valgum or genu varum, suggesting a possible association with hyperlaxity [2]. Although cranial MRI/CT is not the definitive diagnostic criterion, majority of patients with SOTOS exhibit characteristic findings, such as ventricular dilation (particularly in the trigone region), midline anomalies (agenesis or hypoplasia of the corpus callosum, cavum septum pellucidum, and mega cisterna magna), small cerebellar vermis, and cerebral atrophy [23].

*NSD1*, the only gene known to cause SOTOS, encodes the histone methyltransferase protein (H3K36 methylation), which is involved in gene transcription control and as an epigenetic marker [24]. *NSD1* expression has been detected in numerous tissues across diverse organisms. *NSD1* expression levels are upregulated in the pancreas, brain, hematopoietic organs (lymphoid tissues and bone marrow), and the male reproductive tract. *NSD1* gene cloning revealed that*NSD1* expression is evident in multiple tissues, and western blotting assays revealed two transcript variants, attributable to the alternative splicing of exons 3 and 4 [25]. Mutations in several other genes with similar functionality, such as SETD2, DNMT3A, or APC2, can also lead to SOTOS-like syndromes [26–28].

All four cases in our study exhibited clinical and radiographic findings consistent with previous reports, but a definitive diagnosis was highly dependent on genetic techniques. Molecular genetic methods successfully identified the diagnostic variants in the four cases examined in our study, encompassing three sequence variants and one intragenic CNV. To the best of our knowledge, all four variations have not been previously reported. Haploinsufficiency as the pathogenic mechanism of *NSD1*is well established [8]. Case 1 carried a de novo truncating variant, *NSD1*(NM_022455): c.2686 C > T(p.Q896X); Case 2 harbored a *de novo* frame-shift variant, *NSD1*(NM_022455): c.2858_2859delCT(p.S953X), resulting in early translation termination; Case 3 possessed a de novo heterozygous microdeletion, chr5: 5q35.2q35.3(176,516,604–176,639,249)×1, as detected via CMA and encompassing the 5′-UTR fragment of *NSD1*. The variants in Cases 1–3 are null variants that either hinder proper gene expression or yield truncated proteins prone to degradation. Thus, the variants in Cases 1–3 are pathogenic. The missense variant, *NSD1*(NM_022455): c.6397T > G(p.C2133G), in Case 4 resides in the plant homeodomain (PHD) 5 domain of the NSD1 protein, exhibiting considerable conservation across species. PHD acts as a C4HC3 zinc finger-like motif within nuclear proteins that are involved in chromatin modulation and epigenetics [29]. This variant significantly influenced local hydrogen bond formation, potentially compromising the stability of the protein’s three-dimensional structure. Based on these findings and the alignment with the ACMG’s interpretation criteria [18], we designated this variant as “likely pathogenic.” Recent reports demonstrated that *NSD1* mutations result in dysregulated DNA methylation and transcription of bivalent developmental genes in SOTOS [30, 31]. Consequently, transcriptome sequencing and other epigenetic methods are promising tools for the detection of neurodysplasia, especially in patients with negative genomic results [32].

Genetically, all four cases exhibited *NSD1*-associated variations, with the identification of four novel de novo variants specific to each case. The parents of the four cases were wildtype, and thus, future pregnancy for these couples is accompanied with a very low risk of SOTOS recurrence. However, targeted prenatal diagnosis should be recommended for these couples in subsequent pregnancies.

In summary, this study presents a definitive genetic diagnosis for four children exhibiting overgrowth and central nervous system abnormalities. The genetic variants revealed in this study expand the mutation spectrum of *NSD1* associated with SOTOS. Additionally, this research underscores the potential of NGS technology in facilitating the differential diagnosis of neuropathic conditions characterized by nonspecific clinical phenotypes.

**Table 1:** Detection of variation in the four cases

Case no.	Gene*	Genomic alteration	Peptide alteration	Frequency in three databases*	HGMD*/ClinVar* rating	Pathogenicity rating* (evidence)
1	*NSD1* (NM_022455)	c.2686 C > T	p.Q896X	-/-/-	-/-	Pathogenic (PVS1 + PS2 + PM2 + PP3)
2	*NSD1* (NM_022455)	c.2858_2859delCT	p.S953X	-/-/-	-/-	Pathogenic (PVS1 + PS2 + PM2 + PP3)
3	*NSD1* (NM_022455)	5q35.2q35.3(176,516,604–176,639,249)×1	null	-/-/-	-/-	Pathogenic
4	*NSD1* (NM_022455)	c.6397T > G	p.C2133G	-/-/-	-/-	Likely pathogenic (PS2 + PM2 + PP2 + PP3)

*The transcript no. of *NSD1* was NM_022455;

Three databases: 1000 g2015aug_eas, https://www.internationalgenome.org/; ExAC_EAS, http://exac.broadinstitute.org; gnomAD_exome_EAS, http://gnomad.broadinstitute.org/;

HGMD: Human Gene Mutation Database (Professional Version 2019.4, http://www.hgmd.cf.ac.uk/ac/index.php);

ClinVar: https://www.ncbi.nlm.nih.gov/clinvar/;

Pathogenicity rating: In line with the guideline of the American College of Medical Genetics and Genomics (Ref. 18; Richards et al., 2015)

### Electronic supplementary material

Below is the link to the electronic supplementary material.

Supplementary Material 1

## Data Availability

The datasets presented in this study can be found in online repositories. The sequencing results have been deposited to the Figshare repository and can be accessed via the following links: 10.6084/m9.figshare.24135969.

## References

[1] Sotos JF, Dodge PR, Muirhead D, Crawford JD, Talbot NB (1964). CEREBRAL GIGANTISM IN CHILDHOOD. A SYNDROME OF EXCESSIVELY RAPID GROWTH AND ACROMEGALIC FEATURES AND A NONPROGRESSIVE NEUROLOGIC DISORDER. N Engl J Med.

[2] Tatton-Brown K, Cole TR, Rahman N, Sotos Syndrome. 2004 Dec 17 [Updated 2022 Dec 1]. In: Adam MP, Mirzaa GM, Pagon RA, editors. GeneReviews® [Internet]. Seattle (WA): University of Washington, Seattle; 1993–2023. https://www.ncbi.nlm.nih.gov/books/NBK1479/.

[3] Tatton-Brown K, Douglas J, Coleman K, Baujat G, Cole TR, Das S, Horn D, Hughes HE, Temple IK, Faravelli F (2005). Genotype-phenotype associations in Sotos syndrome: an analysis of 266 individuals with NSD1 aberrations. Am J Hum Genet.

[4] Gibson WT, Hood RL, Zhan SH, Bulman DE, Fejes AP, Moore R, Mungall AJ, Eydoux P, Babul-Hirji R, An J (2012). Mutations in EZH2 cause Weaver syndrome. Am J Hum Genet.

[5] Priolo M, Schanze D, Tatton-Brown K, Mulder PA, Tenorio J, Kooblall K, Acero IH, Alkuraya FS, Arias P, Bernardini L (2018). Further delineation of Malan syndrome. Hum Mutat.

[6] Cohen AS, Tuysuz B, Shen Y, Bhalla SK, Jones SJ, Gibson WT (2015). A novel mutation in EED associated with overgrowth. J Hum Genet.

[7] Edmondson AC, Kalish JM (2015). Overgrowth syndromes. J Pediatr Genet.

[8] Kurotaki N, Imaizumi K, Harada N, Masuno M, Kondoh T, Nagai T, Ohashi H, Naritomi K, Tsukahara M, Makita Y (2002). Haploinsufficiency of NSD1 causes Sotos syndrome. Nat Genet.

[9] Kurotaki N, Harada N, Shimokawa O, Miyake N, Kawame H, Uetake K, Makita Y, Kondoh T, Ogata T, Hasegawa T (2003). Fifty microdeletions among 112 cases of Sotos syndrome: low copy repeats possibly mediate the common deletion. Hum Mutat.

[10] Ferilli M, Ciolfi A, Pedace L, Niceta M, Radio FC, Pizzi S, Miele E, Cappelletti C, Mancini C, Galluccio T et al. Genome-Wide DNA Methylation Profiling Solves Uncertainty in Classifying NSD1 Variants. *Genes (Basel)* 2022, 13(11). 10.3390/genes13112163 PMC9690023 36421837

[11] Testa B, Conteduca G, Grasso M, Cecconi M, Lantieri F, Baldo C, Arado A, Andraghetti L, Malacarne M, Milani D et al. Molecular analysis and reclassification of NSD1 gene variants in a cohort of patients with clinical suspicion of Sotos Syndrome. Genes (Basel) 2023, 14(2). 10.3390/genes14020295 PMC9956575 36833222

[12] Ha K, Anand P, Lee JA, Jones JR, Kim CA, Bertola DR, Labonne JD, Layman LC, Wenzel W, Kim HG. Steric clash in the SET domain of histone methyltransferase NSD1 as a cause of Sotos Syndrome and its genetic heterogeneity in a Brazilian cohort. Genes (Basel) 2016, 7(11). 10.3390/genes7110096 PMC5126782 27834868

[13] Foster A, Zachariou A, Loveday C, Ashraf T, Blair E, Clayton-Smith J, Dorkins H, Fryer A, Gener B, Goudie D (2019). The phenotype of Sotos syndrome in adulthood: a review of 44 individuals. Am J Med Genet C Semin Med Genet.

[14] Conteduca G, Testa B, Baldo C, Arado A, Malacarne M, Candiano G, Garbarino A, Coviello DA, Cantoni C (2023). Identification of alternative transcripts of NSD1 gene in Sotos Syndrome patients and healthy subjects. Gene.

[15] Arsham MS, Barch MJ, Lawce HJ (2017). The AGT cytogenetics laboratory manual.

[16] Yang K, Liu Y, Wu J, Zhang J, Hu HY, Yan YS, Chen WQ, Yang SF, Sun LJ, Sun YQ (2022). Prenatal cases reflect the complexity of the COL1A1/2 Associated Osteogenesis Imperfecta. Genes (Basel).

[17] Wang KLM, Hakonarson H (2010). ANNOVAR: functional annotation of genetic variants from next-generation sequencing data. NUCLEIC ACIDS RES.

[18] Richards S, Aziz N, Bale S, Bick D, Das S, Gastier-Foster J, Grody WW, Hegde M, Lyon E, Spector E (2015). Standards and guidelines for the interpretation of sequence variants: a joint consensus recommendation of the American College of Medical Genetics and Genomics and the Association for Molecular Pathology. GENET MED.

[19] Ioannidis NM, Rothstein JH, Pejaver V, Middha S, McDonnell SK, Baheti S, Musolf A, Li Q, Holzinger E, Karyadi D (2016). REVEL: an Ensemble Method for Predicting the pathogenicity of rare missense variants. AM J HUM GENET.

[20] Manor J, Lalani SR (2020). Overgrowth Syndromes-Evaluation, diagnosis, and management. Front Pediatr.

[21] Brioude F, Toutain A, Giabicani E, Cottereau E, Cormier-Daire V, Netchine I (2019). Overgrowth syndromes - clinical and molecular aspects and tumour risk. Nat Rev Endocrinol.

[22] Lane C, Milne E, Freeth M (2017). Characteristics of Autism Spectrum Disorder in Sotos Syndrome. J Autism Dev Disord.

[23] Waggoner DJ, Raca G, Welch K, Dempsey M, Anderes E, Ostrovnaya I, Alkhateeb A, Kamimura J, Matsumoto N, Schaeffer GB (2005). NSD1 analysis for Sotos syndrome: insights and perspectives from the clinical laboratory. Genet Med.

[24] Martin-Herranz DE, Aref-Eshghi E, Bonder MJ, Stubbs TM, Choufani S, Weksberg R, Stegle O, Sadikovic B, Reik W, Thornton JM (2019). Screening for genes that accelerate the epigenetic aging clock in humans reveals a role for the H3K36 methyltransferase NSD1. Genome Biol.

[25] Kurotaki N, Harada N, Yoshiura K, Sugano S, Niikawa N, Matsumoto N (2001). Molecular characterization of NSD1, a human homologue of the mouse Nsd1 gene. Gene.

[26] Luscan A, Laurendeau I, Malan V, Francannet C, Odent S, Giuliano F, Lacombe D, Touraine R, Vidaud M, Pasmant E (2014). Mutations in SETD2 cause a novel overgrowth condition. J Med Genet.

[27] Almuriekhi M, Shintani T, Fahiminiya S, Fujikawa A, Kuboyama K, Takeuchi Y, Nawaz Z, Nadaf J, Kamel H, Kitam AK (2015). Loss-of-function mutation in APC2 causes sotos Syndrome features. Cell Rep.

[28] Tlemsani C, Luscan A, Leulliot N, Bieth E, Afenjar A, Baujat G, Doco-Fenzy M, Goldenberg A, Lacombe D, Lambert L (2016). SETD2 and DNMT3A screen in the sotos-like syndrome French cohort. J Med Genet.

[29] Romero VI, Arias-Almeida B, Aguiar SA (2022). NSD1 gene evolves under episodic selection within primates and mutations of specific exons in humans cause Sotos syndrome. BMC Genomics.

[30] Brennan K, Zheng H, Fahrner JA, Shin JH, Gentles AJ, Schaefer B, Sunwoo JB, Bernstein JA, Gevaert O (2022). NSD1 mutations deregulate transcription and DNA methylation of bivalent developmental genes in Sotos syndrome. Hum Mol Genet.

[31] Hamagami N, Wu DY, Clemens AW, Nettles SA, Li A, Gabel HW (2023). NSD1 deposits histone H3 lysine 36 dimethylation to pattern non-CG DNA methylation in neurons. Mol Cell.

[32] Lecoquierre F, Quenez O, Fourneaux S, Coutant S, Vezain M, Rolain M, Drouot N, Boland A, Olaso R, Meyer V (2023). High diagnostic potential of short and long read genome sequencing with transcriptome analysis in exome-negative developmental disorders. Hum Genet.

