# Supplementary material for: Identification of Novel NSD1 variations in four Pediatric cases with sotos Syndrome
Source: BMC Med Genomics. 2024 Apr 29;17:116. doi: 10.1186/s12920-024-01889-5 (PMC11059674; doi:10.1186/s12920-024-01889-5)
Supplement: Supplementary file 1 — Supplementary Material 1 [file 12920_2024_1889_MOESM1_ESM.docx]

**Table S1. PCR Primers for** **RT-PCR**

| **Primers** | **Sequence** | **Length** | **Tm** |
| --- | --- | --- | --- |
| *NSD1*-P1-F | CCAGAGAGTAGACACGGTGC | 123 | 60℃ |
| *NSD1*-P1-R | TGGGAGAAGGGCTGCTTTTT |  |  |
| *NSD1*-P2-F | CGGTCAGAGAAGAAACGCCT | 120 | 60℃ |
| *NSD1*-P2-R | CTTGTGCACCTGCTCCTGTA |  |  |
| β-globin-QF | ACACAACTGTGTTCACTAGC | 110 | 60℃ |
| β-globin-QR | CAACTTCATCCACGTTCACC |  |  |

**Reaction reagents and condition**

The *NSD1* gene copy number was assessed by real-time Quantitative Fluorescence PCR using SYBR Premis Ex Taq II (Perfect Real Time) (Takara) with ABI 7500 system. Data are presented as mean ± standard deviation of three independent real-time PCR experiments. The PCR cycle was as follows: 10 min 95°C, 1 cycle; 10 s 95°C, 30 s 60°C＋fluorescence acquisition, 55 cycles. Values for each gene were normalized to expression level of beta-actin gene (ACTB) via the 2^-ΔΔCT^ method.
